# Delayed publication of clinical trials in cystic fibrosis^☆^

**DOI:** 10.1016/j.jcf.2011.08.004

**Published:** 2012-01

**Authors:** M.N. Hurley, A.P. Prayle, A.R. Smyth

**Affiliations:** a Division of Child Health, School of Clinical Sciences, University of Nottingham, Nottingham, NG7 2UH, UK; b Cochrane Cystic Fibrosis and Genetic Disorders Group, UK

**Keywords:** Cystic fibrosis, Publication bias, Clinical trials

## Abstract

**Background:**

When the publication of important trial data is delayed, or data are never published, this will prevent the proper practice of evidence based medicine through robust systematic reviews. Clinical trial registries allow researchers to interrogate the trial protocol and afford the opportunity to identify studies that have been completed and so determine the time lag between completion and publication.

**Methods:**

We searched ClinicalTrials.gov with the keywords ‘cystic fibrosis’. Intervention trials which had completed 1st Jan 1998–31st Dec 2010 were selected. Time to publication in a peer-reviewed journal was calculated. Survival analyses using the log rank test were undertaken.

**Results:**

We identified 142 records. Of these, 62 had full paper publications. The median time to publication was 3.25 years. Phase of study (phase one studies more delayed, *p* = 0.024) but not source of funding (*p* = 0.34) was associated with time to publication.

**Conclusions:**

Clinical trials in cystic fibrosis take a considerable amount of time to report their findings. More importantly, a large number of trials fail to report at all.

## 1 Introduction

Evidence based medicine depends upon access to the results of well designed trials which have minimal bias [2]. To fully understand the effect of an intervention, one should consider all the available evidence. Without this, conclusions drawn from the literature, upon which clinical decisions rest, may be erroneous. Unfortunately, not all trial results are published. Failure to publish data in a peer-reviewed journal can occur for several reasons, including editorial decisions, difficulties in publishing “negative results” or because the investigator or sponsor has not submitted the results. Thus all the relevant trial data are often not available to clinicians, systematic reviewers, health policy decision makers and patients.

Until recently, the number of unpublished trials was unknown, as there were no registries of conducted trials. However, in 2005 the International Committee of Medical Journal Editors announced that trial publication in participating journals would be dependent upon registration of a summary protocol of the trial with a public registry [3]. This allows an assessment to be made of the total number of clinical trials in any particular field, and additionally allows comparison of the trial report with a protocol summary. Given that registry entries give a completion date of each trial, registries allow the calculation of the time taken from completion of a clinical trial to publication in a peer-reviewed journal.

There is bias in the medical literature, and this can have an impact on the findings of systematic reviews [4]. Time-lag bias has been documented in other areas of clinical practice, such as pharmacology of antidepressants in children [5]. Studies with positive results are published more quickly than other studies [6]. Although it is the most common inherited life limiting disease in the Caucasian population, cystic fibrosis (CF) is a rare disease. Thus the total CF trial literature can be assessed. We present an evaluation of time taken to report clinical trials which involved patients with CF.

## 2 Methods

We searched ClinicalTrials.gov (search date 10th Jan 2011) with the key words ‘cystic fibrosis’, and selected the subgroup of intervention trials which had a recorded completion date between 1st Jan 1998 and 31st Dec 2010. The output from a ClinicalTrials.gov search can be downloaded in a spreadsheet format, which formed the basis for the subsequent analysis. Using details from the registry record of each trial (trial ID, sponsor, trial name and trial investigators) we searched online (using PubMed, Google Scholar and the Cochrane Central Register of Clinical Trials) for the first publication relating to that trial.

Where we could not identify a publication in a peer-reviewed journal, we contacted the listed investigator in the ClinicalTrials.gov record, to determine if the trial had been published. Investigators were not always listed in the record, in which case we contacted the study sponsor.

We categorised each trial by the funding source (Industry, Mixed e.g. Industry/Charity collaboration, National Institutes of Health/U.S. Government, or “other”); phase of study; geographic location of study (single centre *vs.* multi-centre and national *vs.* international study). A script was written which automated the extraction of these data from ClinicalTrials.gov for each trial in the dataset. Number of centres and countries in a study are not part of the standard downloaded spreadsheet and so we could not analyse this variable.

The primary endpoint for our analysis was the time to first publication of a trial report in a peer-reviewed journal from completion of the study. We used the ‘primary completion date’ (recorded on ClinicaTrials.gov) as the time when the study completed. Where this field was not completed we used the ‘completion date’. Where both fields were missing the trial was excluded from the analysis. We conducted a survival analysis for the whole cohort of studies, and additionally several subgroup analyses using the log rank test to determine if the categorical variables funding source, phase of study, number of countries, age of participants and number of sites were associated with the likelihood of publication. We used a Cox proportional hazard model to investigate the effects of the enrolment size upon time to publication. We took statistically significant results from these univariate analyses and combined into a Cox proportional hazards multivariate model to confirm that they remained predictors of time to publication.

Statistical analysis was undertaken with R version 2.13.1 [7].

## 3 Results

We identified 142 study records on ClinicalTrials.gov which met the inclusion criteria. The search of online databases found that 59 of these had publications in a peer-reviewed journal. We contacted the investigators for the remaining studies, and received 29 responses. This identified a further 3 studies which had been published. We did not count 13 studies which had been published in abstract form only. A summary of the included trial characteristics is shown in Table 1.

**Fig. 1:**
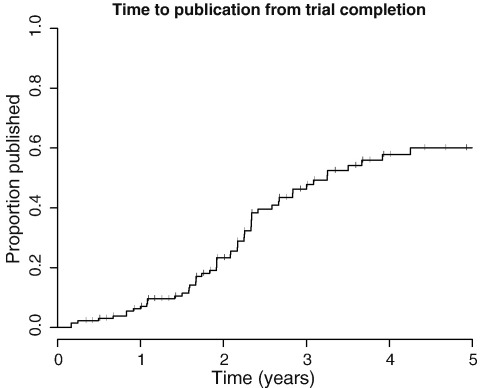
Survival graph showing time to publication (in a peer-reviewed journal) of all studies within the dataset. Vertical bars indicate right censored studies (those which had not published at the end of the study period on the 11th Jan 2011 when the ClinicalTrials.gov search took place).

Overall, 62 (43.7%) completed studies have published in a peer-reviewed journal. However this value considers all studies equally, irrespective of time since study completion. For example, for ten unpublished studies there was less than 365 days of follow up, so the overall rate of 43.7% publication underestimates actual publication. We conducted a survival analysis, using the endpoint of publication in a peer-reviewed journal, and calculated cumulative publication percentage over a 5-year period for all studies, right censoring studies which had not published at the time of the search (Fig. 1). Records of the completion date or primary completion date were missing for 10 studies, which were excluded from the analysis. The median time to publication of all studies was 3.25 years. As can be seen in Fig. 1, a large proportion failed to publish within 5 years.

**Fig. 2:**
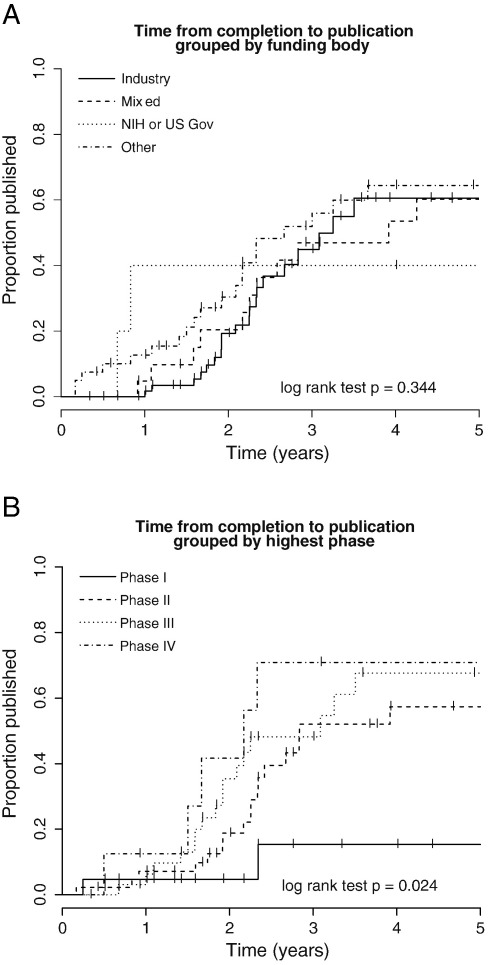
A. Survival graph showing time to publication of studies, categorised according to funding body listed on ClinicalTrials.gov. There were only 5 included studies in the “NIH or US Gov” group. There was no significant difference between groups in the proportion of studies which published within the follow up period. B. Survival graph showing time to publication of studies grouped by study Phase. Phase I studies were least likely to publish results. Vertical bars on each plot indicate right censored studies.

We conducted survival analyses (log rank test) with the studies grouped by funding source and study phase, (e.g. I, II, III, or IV) (see Fig. 2A and B and Table 2). We found no evidence that the funding source influences the time to publication (*p* = 0.34; median times to publication were: Industry 3.25 years, Mixed 3.92 years, NIH or US Gov 9.92 years, Other 2.67 years, see also Table 2). Analysis of time to publication grouped by phase of study suggested that study phase influences timing of publication (*p* = 0.024; median times to publication were as follows: Phase I < 50% published, Phase II 2.84 years, Phase III 3.08 years, Phase IV 2.17 years). Examination of the survival graphs suggests that Phase I studies take longer and are less likely to publish compared to the later phase studies (Fig. 2B). We found no evidence that international studies are more likely to publish compared to single nation studies (log rank *p* = 0.20), no evidence that multi-site trials are more likely to publish compared to single centre studies (log rank *p* = 0.99; data on number of sites were not available for 20 studies which were excluded from this analysis), and no evidence that age-group of participants influenced likelihood of publication (log rank *p* = 0.95). In a univariate Cox proportional hazard analysis, studies with a larger number of participants enrolled were more likely to publish more quickly (*p* = 0.00565). This enrolment was no longer significant (*p* = 0.0510) in the multivariate model including study phase (the other significant variable identified in the univariate analysis).

## 4 Discussion

We have found that on average it takes 3.25 years between completion of a clinical trial in cystic fibrosis and the publication of the trial report in a peer-reviewed journal. Importantly a large proportion of studies fail to report within 5 years of study completion. We have also found evidence that Phase I studies are less likely to report.

It was surprisingly difficult in some cases to match clinical trial reports with the record on ClinicalTrials.gov. Often the title of the study is different, and the lead investigator is not always an author on the final peer-reviewed trial report. Although some trial reports give the trial ID number in the abstract or as a footnote to the paper, this is not universal.

In a recent systematic review of studies of time to publication of clinical trial results, the two included studies investigated either the time of granting of ethical approval for the study or the time of first patient enrolment [6]. In this systematic review, studies with positive results were reported within 4–6 years, and negative results within 6–8 years. In our study, the median time for publication was 3.25 years, however this did not include the time taken to actually conduct the trial. We reasoned that some trials may require a longer period of follow up, or may take longer to recruit patients with a rare disease, so we preferred to measure the time to publication in terms of the interval after the study had completed.

We made great efforts to try to ensure that we have found all clinical trial reports, published in a peer-reviewed journal. In addition to using 3 search engines to locate trial reports, we also contacted either the lead investigator or (where there was no record of the investigator) the sponsor of the study. We believe that we have found all the published studies. We present the data through a survival analysis, which also allowed robust statistical comparison between specific subgroups.

We did not use registries other than ClinicalTrials.gov (such as the ISRCTN or the WHO trial registry). Neither EudraCT nor ISRCTN includes a completion date. Recent additions to ClinicalTrials.gov such as the facility to upload summary study results will also make ClinicalTrials.gov a valuable resource for research into publication bias in the future [8].

We are reliant upon the study investigators and sponsors for maintaining accurate records on ClinicalTrials.gov. For example, if a trial completed early or late, and the ClinicalTrials.gov record was not updated, this would introduce error into our calculated time to publication. We have no way of knowing if a trial actually completed on the date listed on the trial record.

The response rate after contacting study investigators and sponsors was low. We received 29 responses which lead to us identifying a further 3 studies which our initial search had not located. Although a large proportion of individuals and institutions who were contacted did not respond, in our opinion investigators who have published their work are likely to reply with the details of the publications. We hope therefore to have found all the relevant publications which fall within our inclusion criteria.

When patients enter into clinical trials, they do so with the altruistic expectation that this will help others (and perhaps themselves) in the future. Dissemination of the data from the trial in a peer-reviewed clinical trial report which complies with CONSORT guidelines represents the 'gold-standard' in reporting data from clinical trials, and gives the best chance of carefully considering the data which the trial has generated. This is essential to making evidence based decisions. We acknowledge that the complexity of many clinical trials means that it can take some time from trial completion to the submission of the clinical trial report.

We have found that studies take a considerable amount of time to report. More importantly, a large number of studies fail to report at all. Delaying publication (or failing to publish) at best results in delaying the adoption of an improvement in practice, and at worst results in inadvertently replicating clinical research or skewing the evidence base such that the wrong conclusions are made. In our opinion the patients who contribute time, effort (and expose themselves to a degree of risk) by taking part in clinical trials deserve to know that the results of the trials, in which they take part, will be published.

## Conflict of interest statement

MH and AP have no conflicts to declare. AS has provided consultancy for Mpex Pharmaceuticals. He has also been involved in the following industry trials:Boehringer Ingelheim*,* Ltd. (*UK*)—inhaled tiotropium as a long acting bronchodilator in CF.

## Figures and Tables

**Table 1:** Characteristics of included studies.

Characteristic	Subgroup	*n* (%)
Age range		
	Adult	38 (26.6%)
	Adult and paediatric	69 (48.4%)
	Paediatric	21 (14.8%)
	Missing data	14 (10.2%)
Funding source		
	Industry	68 (47.9%)
	Mixed	27 (19.0%)
	NIH or US Gov	7 (4.9%)
	Other	40 (28.2%)
Phase of study		
	I	26 (18.3%)
	II	46 (32.4%)
	III	34 (23.9%)
	IV	9 (6.3%)
	Unclassified	27 (19.1%)
Number of centres		
	Multi-centre	60 (42.3%)
	Single centre	62 (43.7%)
	Missing data	20 (14.1%)
Number of nations		
	Multinational	121 (85.2%)
	Single nation	21 (14.8%)
No. of study participants		
	Median	30
	Interquartile range	18–74
	Range	3–517

**Table 2:** Percentage Publication Rate by Funding Source. Publication rates for the first 5 years following completion. Percentages refer to cumulative proportion of studies published by year for each group. *n*, numbers represent studies for which the time to publication data were available. For 10 studies these data were not available. The total numbers in each category were as follows: Industry = 68, Mixed = 27, NIH or US Gov = 7, Other = 40.

Year	Industry (*n* = 65)	Mixed (*n* = 22)	NIH or US Gov (*n* = 5)	Other (*n* = 40)
1	1.7	4.8	40	12.7
2	19.3	20.4	40	30.4
3	44.9	46.9	40	51.9
4	60.5	53.6	40	64.4
5	60.5	60.2	40	64.4
